# Effects of common litter types and their physicochemical properties on the welfare of broilers

**DOI:** 10.14202/vetworld.2022.1523-1529

**Published:** 2022-06-20

**Authors:** Tarek Boussaada, Kaouthar Lakhdari, Salha Amira Benatallah Samira Meradi

**Affiliations:** Scientific and Technical Research Centre for Arid Areas (CRSTRA), Biskra, Algeria

**Keywords:** broiler, litter quality, litter types, physicochemical characteristics of litter, welfare indicators

## Abstract

**Background and Aim:**

In broiler production, the poor quality litter not only may lead to a deterioration of the welfare status but also negatively affect carcass quality, overall health and growth performance, which may result in economic losses. The effects of litter types on the welfare of broilers are known but the effects of their characteristics have been little studied. This study aimed to evaluate correlations between welfare parameters of broilers and physicochemical characteristics of five common litter types.

**Materials and Methods:**

Over 42 days, 600 (Cobb 700) male broiler chicks were placed within 30 pens (each 2 m^2^) at a density of 10 birds/m^2^. The experiment included five treatments with six replicates per treatment. The following litter (or bedding) materials were examined: Standard quality straw, low-quality straw, wood shavings (WS), sawdust, and crop residues. Footpad condition, hock burns, and plumage cleanliness, as well as litter condition, were scored according to previously developed point scale systems. Litter quality was evaluated according to pH level, moisture, water-holding capacity, and ammonia content.

**Results:**

No significant differences were found among litter types in terms of pH, moisture content, or ammonia levels. WS had a significant positive effect on footpad health and plumage cleanliness. However, hock burn was not affected by different bedding types. The severity of pododermatitis was negatively correlated with litter type (r = −0.78; p < 0.001) and positively correlated with the litter scores (r = 0.67; p < 0.001). However, contact dermatitis observed (pododermatitis and hock burn) was not correlated with any of the physicochemical parameters we studied. Meanwhile, we observed a correlation between footpad lesions and hock burn (r = 0.45; p < 0.05), and between footpad lesions and plumage cleanliness (r = 0.59; p < 0.01).

**Conclusion:**

For all litter types examined, contact dermatitis was not correlated with any of the physicochemical components we studied. There were, however, significant correlations between litter type and footpad lesions, as well as between footpad dermatitis and hock burns.

## Introduction

The selection of litter types can play an important role in the raising and farming of broilers due to their influence on the growth performance, carcass quality, overall health, and welfare of broilers [1]. Broiler welfare depends on litter and air quality since broilers spend the majority of their lives in contact with litter material, including their feet, legs, and abdomens. Fast-growing broilers, especially after 3 weeks of age, spend much time lying down. Further, the choice of litter material may affect ammonia levels, litter moisture content, and the prevalence of air dust in poultry houses [2]. For example, the physicochemical characteristics of litter, such as water-holding capacity and absorbance capacity, can affect litter conditions during rearing periods [3]. It is well known that many factors can deteriorate litter quality, such as the type and amount of litter material used, feed composition, litter management techniques, housing type, ambient conditions, drinker management, health status, stocking density, and slaughter age [4–8].

Footpad condition is affected by a number of factors [9]; however, the type of bedding material used and the amount of moisture in the litter have both been identified as critical contributors in the development of footpad dermatitis (FPD). FPD can have substantial welfare and economic consequences [10]. Litter wetness has been considered to be the main factor contributing to the appearance of footpad lesions in broilers, turkeys, and laying hens [11, 12]. Meluzzi *et al*. [13] showed a high positive correlation (r = 0.89) between litter wetness and footpad dermatitis in broilers. Wu and Hocking [14] also found that wet litter (more than 30% moisture) leads to impaired footpad condition. Moreover, wet litter conditions lead to deterioration in plumage cleanliness [15], broiler growth, and feed efficacy [10].

Kaukonen *et al*. [16] found that broilers from houses with higher litter ammonia content and lower pH had better footpad quality. Ammonia has not been found to have an effect on footpad dermatitis in several studies [11, 12, 17]. The presence of high levels of ammonia in the litter promotes irritation of the birds’ eyes and larynges, as well as an increase in mortality rates [9].

Most published studies assessing litter quality in broiler houses have been based on the effects of litter types on contact dermatitis [6, 18–20]. However, none of these authors have investigated correlations between these lesions and characteristics of litter material.

In our previous study, we found that the lesions of pododermatitis appeared from the first week of the broiler chicks reared on five litter types [21]. However, at this age, the different types of litter still seem dry and crumbly. In view of these results, it might be interesting to identify key parameters which could contribute to the appearance of contact dermatitis. This study aimed to evaluate correlations between welfare parameters of broilers and physicochemical characteristics of five common litter types.

## Materials and Methods

### Ethical approval

The experiment has been approved by the scientific and technical research center on arid regions, Biskra (Algeria). All experimental procedures were complied with the European Union Directive on Legislation for the protection of animals used for scientific purposes, 2010-63-EU.

### Study period and location

The study was conducted during September and October 2021 at a private broiler farm in Kaïs, Khenchela, Algeria. Laboratory analyses were performed in the Laboratory of Scientific and Technical Research Centre for Arid Areas (CRSTRA), Algeria.

### Design and husbandry

The experiment was conducted with six hundred 1-day-old male broiler chicks (Cobb 700). Chicks were randomly allocated into five treatments (WS, S, standard quality straw (SQS), low-quality straw (LQS), and crop residues [CR]) of six replicates pens of 20 birds (10 birds/m^2^) from 1 to 42 days of age. Depth of the litter was 5 cm. Each pen measured 2 m^2^ was contained nipple drinkers and feeders. Throughout the experiment, both feed and water were *ad libitum*. The three phases of the commercial feeding program were starter (1–15 days), grower (16–30 days), and finisher (31–42 days). All diets were formulated to meet National Research Council recommendation [22].

### Litter sample collection

Litter samples were collected at the end of the experiment from three different locations per pen in plastic containers, with areas around drinkers and feeders avoided, using the method described by Smalberger and van Rensburg [23].

### Litter condition and quality assessment

Litter condition was scored weekly according to the method described by Litt *et al*. [24]. Moreover, each litter sample collected at the end of the experiment was thoroughly mixed and subsequently analyzed. Litter quality was assessed by means of moisture, water-holding capacity, pH, and ammonia levels. Moisture content was determined after drying at 105°C for 24 h. The pH value was determined using an electronic meter (WTW Multi 350 i) as follows: A 30 g of litter sample was macerated in a beaker, then 250 mL deionized water were added, agitated for 5 min and read with a pH meter after left to rest for 30 min.

The methodology proposed by Garcês *et al*. [1] was used to determine the water-holding capacity, as follows: Each sample of litter was dried until constant weight and 50 g of litter was placed in a 500 mL beaker; the beaker was filled with water and left to stand for 30 min; the sample was drained for 3 min, and it was weighed again; the percentage of water absorbed was calculated on a dry matter basis.

Litter NH_3_ emissions were determined with the micro-diffusion method described by Hernandes and Cazetta [25], as follows: 100 g of litter sample was weighed and leveled in a 500 mL cylindrical flask; a 50 mL beaker containing 10 mL of 2% (m/v) boric acid was placed on top of the litter; the flask was closed and incubated for 20 h at 30°C; the boric acid solution was then titrated against sulfuric acid 0.1 N with metal orange and bromocresol green; the amount of sulfuric acid used (A) was multiplied by its normality and the molecular weight of ammonia to calculate volatilized NH_3_ (in mg/100 g of litter):

NH_3_ = A × 0.1 × 17

### Welfare indicators

A welfare assessment was performed on the birds after 42 days of the experiment. The birds were scored on three main welfare measures: Footpad dermatitis, hock burn, and plumage cleanliness. The incidence and severity of FPD were measured by the method described by Michel *et al*. [26], using a scoring range from 1 to 5, with 1 representing no lesion and five extreme lesions. The evaluation was performed on both feet. For hock burns, the evaluation was performed according to the method of Bignon *et al*. [3], scoring from 1 to 3. No lesions was equal to score 1, <25% hock area was equal to score 2, and more than 50% of the hock area was equal to score 3. Breast plumage dirtiness of birds was scored visually from 0 (very clean) to 3 (very dirty) as reported by De Jong *et al*. [27].

### Statistical analysis

Statistical analysis was performed using the software package Statistical Package for the Social Sciences (SPSS Version 22.0, IBM Corp., NY, USA). All measured parameters were tested for normality by calculating the Kolmogorov–Smirnov test statistic, and all followed a normal distribution. Welfare indicators and litter quality data were analyzed using general linear models. The correlations between the welfare parameters and characterization of litter materials were tested with Spearman’s rho test. Statistical significance was set at p < 0.05.

## Results

### Welfare indicators

Broiler welfare status was evaluated by assessing scores of footpad dermatitis, hock burn, and plumage cleanliness at the age of 42 days. These welfare indicator scores are summarized in Tables-1 and 2. The effect of litter type on average scores of footpad condition, hock burns, and plumage cleanliness (on 42 days) is shown in Table-1, which shows the best scores occurred when WS were used as bedding. Litter type had a significant effect on footpad condition (p < 0.001) and plumage cleanliness (p = 0.032). However, hock burn was not significantly affected by bedding type.

**Table 1:** Effects of litter type on average score of footpad, hock burns, and plumage cleanliness in broilers at 42 days of age.

Litter type	Mean footpad score	Mean hock burn score	Mean cleanliness score
SQS	3.42 ± 0.45^c^	2.73 ± 0.28	2.92 ± 0.08^c^
LQS	3.53 ± 0.42^c^	2.63 ± 0.23	2.95 ± 0.05^c^
CR	3.03 ± 0.23^b,c^	2.62 ± 0.26	2.88 ± 0.10^b^
S	2.28 ± 0.53^a,b^	2.60 ± 0.26	2.70 ± 0.25^a^
WS	2.12 ± 0.55^a^	2.15 ± 0.66	2.67 ± 0.29^a^
p-value	p<0.001	NS	p = 0.032

^a.b,c^ Means in the columns within the same treatment with different superscripts differ significantly (p<0.05).

SQS = Standard quality straw, LQS = Low-quality straw, CR = Crop residues, S = Sawdust, WS = Wood shaving

**Table 2:** Scoring percentages of footpad lesions, hock burn, and plumage cleanliness in broilers at 42 days of age.

Litter type	Frequency of footpad score (%)					Frequency of hock burn score (%)			Frequency of plumage cleanliness (%)			
		
	1	2	3	4	5	1	2	3	0	1	2	3
SQS	0^a^	1.7^a^	38.3	60^c^	0	0	26.7	73.3	0	0	8.3^a,b^	91.7^c^
LQS	1.7^b^	3.3^a^	35	60^c^	0	0	36.7	63.3	0	0	5^a^	95^c^
CR	0^a^	13.3^b^	70	16.7^b^	0	0	38.3	61.7	0	0	12^b^	88.3^b^
S	23.3^c^	31.7^c^	38.3	6.67^a,b^	0	0	40	60	0	0	30^c^	70^a^
WS	23.3^c^	45^d^	28.3	3.33^a^	0	25	35	40	0	0	33^c^	66.7^a^
p-value	0.006	<0.001	NS	0.001	NS	NS	NS	NS	NS	NS	0.037	0.037

^a,b,c^ Means within a column without a common superscript are different (p<0.05). Footpad: Score 1 = No or type I lesion, Score 2 = Type II lesion of size<50%, Score 3 = Type II lesion of size>50%, Score 4 = Type III lesion of size<50%, and Score 5 = Type III lesion of size>50; Hock burns: Score 1 = No lesion, Score 2 = <25% hock area, and Score 3 = More than 50% hock area; Plumage cleanliness: Score 0 = Completely clean, Score 1 = Slight dirtiness, Score 2 = Moderate dirtiness, and Score 3 = Extensive dirt. SQS = Standard quality straw, LQS = Low-quality straw, CR = Crop residues,

S = Sawdust, WS = Wood shaving

The scoring percentages of footpad lesions, hock burn, and plumage cleanliness in broilers on 42 days of age are shown in Table-2. The prevalence of footpad dermatitis was influenced by litter types for scores 1, 2, and 4. The majority of broilers reared on both straw litter types (60%) displayed severe footpad lesions (score 4). While, the prevalence of severe footpad dermatitis (score 4) was lowest on WS (3.3%) compared with other types of litter.

The evaluation of hock burns on 42 days of age showed that the occurrence and severity of hock burns were not influenced by different litter types (p > 0.05). However, the appearance of severe hock burn (score 3) was lower in broilers reared on WS (40%) compared to other litter types.

For plumage cleanliness, litter types had no influence on the distribution of scores 0 and 1; however, litter type affected the percentage of plumage cleanliness scores 2 and 3 (p = 0.037). In general, animals showed a continuous decrease in plumage cleanliness toward the end of the rearing period, with the lowest scores in broilers reared on WS (mean score = 2.67 ± 0.29) and the highest in these reared on LQS (mean score = 2.95 ± 0.05).

### Litter quality assessment

Overall, the average litter score increases with the age of the birds (p < 0.001) (Table-3). At the end of the experiment, the score was the worst on low straw quality (4.8). The best results were achieved for S (3.2), followed by WS (3.3), SQS (4.0), and CR (4.2).

**Table 3:** Litter quality assessment.

Litter type	Litter scoring						p-value

	Week 1	Week 2	Week 3	Week 4	Week 5	Week 6	
SQS	2.2 ± 0.4^b^	2.7 ± 0.5^b^	3.7 ± 0.5^c^	4.0 ± 0^b^	4.0 ± 0^b^	4.0 ± 0^b^	<0.001
LQS	3.0 ± 0^c^	3.0 ± 0^b^	4.0 ± 0^c^	4.0 ± 0^b^	4.0 ± 0^b^	4.8 ± 0.4^c^	<0.001
CR	1.8 ± 0.4^b^	2.5 ± 0.5^a,b^	3.5 ± 0.5^b,c^	3.8 ± 0.4^b^	4.2 ± 0.4^b^	4.2 ± 0.4^b^	<0.001
S	1.2 ± 0.4^a^	2.0 ± 0^a^	2.8 ± 0.4^a,b^	3.2 ± 0.4^a^	3.0 ± 0^a^	3.2 ± 0.4^a^	<0.001
WS	1.2 ± 0.4^a^	2.0 ± 0^a^	2.2 ± 0.4^a^	3.0 ± 0^a^	3.0 ± 0^a^	3.3 ± 0.5^a^	<0.001
p-value	<0.001	<0.001	<0.001	<0.001	<0.001	<0.001	

^a,b,c^ Means within a column without a common superscript are different (p<0.05). SQS = Standard quality straw, LQS = Low-quality straw, CR = Crop residues, S = Sawdust, WS = Wood shaving

### Physicochemical characteristics of litter

The physicochemical characteristics of the different litter materials evaluated at the end of the experiment are shown in Table-4. On 42 days, the average moisture content of different types of litter in the present study was between 55 and 60%, with no significant differences among all groups (p > 0.05). WS had the lowest litter moisture levels (55.36%), while CR showed the highest moisture levels (59.14%). The type of litter material did not affect the litter pH (p > 0.05), which was relatively homogeneous in all the treatment groups (9.17 < pH < 9.32). Furthermore, litter material did not seem to affect the litter ammonia (p > 0.05). However, of all the bedding materials tested, LQS showed the highest water-holding capacity (p = 0.038).

**Table 4:** Physicochemical properties of different litter materials on 42 days.

Litter type	WHC (g of H_2_ O/g)	Moisture (%)	pH	NH_3_ (mg/100 g)
SQS	3.32^c^	57.97	9.17	3.97
LQS	3.20^b^	55.61	9.16	3.26
CR	3.18^b^	55.36	9.32	4.08
S	2.95^a^	59.14	9.17	3.91
WS	3.13^b^	56.46	9.27	3.15
SEM	0.06	0.72	0.03	0.19
p-value	p = 0.038	NS	NS	NS

^a,b,c^ Means within a column without a common superscript are different (p<0.05). WHC = Water-holding capacity (DM basis). SEM: Standard error of the mean, SQS = Standard quality straw, LQS = Low-quality straw, CR = Crop residues, S = Sawdust, WS = Wood shaving

### Correlation between welfare indicators and physicochemical characteristics of litter types

The correlation between welfare indicators (pododermatitis, hock burn, and plumage cleanliness) and physicochemical characteristics of the various litter types evaluated at the end of the experiment is shown in Table-5. We found a correlation between footpad lesions and hock burn (r = 0.45; p < 0.05), and between footpad lesions and plumage cleanliness (r = 0.59; p < 0.01). The severity of pododermatitis was negatively correlated with litter type (r = −0.78; p < 0.001) and positively correlated with the litter scores (r = 0.67; p < 0.001). However, levels of contact dermatitis (pododermatitis and hock burn) were not correlated with any of the physicochemical characteristics of litter measured in this study.

**Table 5:** Spearman correlation between welfare indicators and physicochemical characteristics of litter.

Variable	Pododermatitis	Hock burn	Plumage cleanliness	Litter types	Litter scores	pH	NH_3_
Hock burn	0.45*	-	-	-	-	-	-
Plumage cleanliness	0.59**	0.34	-	-	-	-	-
Litter types	−0.78**	−0.25	−0.40*	-	-	-	-
Litter scores	0.67**	0.10	0.39*	0.65**	-	-	-
pH	−0.27	−0.10	0.07	0.23	−0.21	-	-
NH_3_	−0.23	−0.25	−0.39*	0.04	−0.02	−0.23	-
Moisture	−0.03	0.14	0.04	−0.06	0.001	−0.16	0.33

* p<0.05,

** p<0.01

## Discussion

### Welfare indicators

The occurrence and severity of pododermatitis and hock burn are effective indicators for assessing the welfare of broilers. In the present study, the best average scores of footpad condition were recorded in the birds reared on WS (2.12 ± 0.55). This is in agreement with the previous studies, which have shown better footpad health for animals kept on WS compared to straw litter [13, 28–31]. Furthermore, the higher FPD scores in broilers raised on both straw types (LQS and SQS) support previous observations made by Bilgili *et al*. [6].

Usually, litter characteristics linked to the appearance of footpad lesions can also cause other forms of lesions, such as hock burns and breast blisters [32, 33]. However, even under conditions favorable for their development, hock burns develop slowly, as confirmed by Škrbić *et al*. [31]. Furthermore, broilers in our study showed a continuous decrease in hock health at the end of the fattening period (data not tabulated). A deterioration in hock condition over the rearing period could be explained by weight gain [34] and reduced physical activity [35–37], resulting in broilers sitting in direct contact for longer periods with litter with higher moisture content [36]. We observed no effect of litter type on hock burns, confirming the findings of Kuleile *et al*. [38].

We observed that plumage cleanliness decreased over time for all litter types (data not tabulated). Broilers reared on WS had the best plumage cleanliness score. However, litter types did not affect the distribution of scores 0 and 1 (p > 0.05); but litter type affected the percentage of plumage cleanliness scores 2 and 3 (p = 0.03). Kaukonen *et al*. [39] studied the effects of bedding materials and elevated platforms on contact dermatitis and plumage cleanliness of broilers and showed that plumage cleanliness score was not affected by litter material, platform treatment, or farm. Li *et al*. [40] and Çavuşoğlu and Petek [20] found that broilers reared on litter and a completely perforated flooring system had a decline in feather cleanliness with time.

### Litter quality

Many factors can influence the quality of litter material, such as the type of material used, the thickness of the bedding layer, temperature, ventilation, stocking density, and broiler nutrition [4–8]. Deteriorated litter quality negatively affects footpad dermatitis and hock burns in broiler chickens [41] and broiler turkeys [12]. In the present study, average litter quality decreased over time. Haslam *et al*. [32] reported that the excretion of feces due to different types of disease or increases in the water consumption of birds may deteriorate the quality of litter. As a result, slower growth and higher mortality lead to lower stocking densities and a higher incidence of hock burns.

### Physicochemical characteristics of litter

We found moisture levels to be lower when WS were used as bedding material compared to the other litter types. Similar findings were reported previously by Škrbić *et al*. [31], who compared the litter moisture of WS with chopped straw. By contrast, another previous study found WS to have higher moisture content than either sand or paper [42].

An ideal litter material should not only be able to absorb moisture, but should also release moisture quickly. In our study, litter type did not significantly affect the overall moisture at the end of the rearing period. However, we found water-holding capacity to be significantly higher in straw treatments than WS, CR, and S (p = 0.038). These results agree with that of Farhadi [43], who also compared the water-holding of wheat straw with WS and rice hulls.

In the previous studies [44, 45], litter moisture was affected by management practices and housing conditions (temperature, relative humidity, and ventilation) but also factors such as the water-holding and water-releasing capacity of the litter material [6, 46].

Litter moisture is considered an important factor affecting the lesions of pododermatitis [11, 32]. However, some studies found that litter moisture did not directly affect the incidence of footpad dermatitis lesions, but rather there are many other contributing factors [31]. Litter ammonia is another important factor in the appearance of FPD lesions [47]. The low quantities of NH_3_ observed in our experiment could be related to the high moisture content of the litter material used, as demonstrated by Garcês *et al*. [1]. On the other hand, results confirm that the best footpad status observed in broiler reared on litter with higher ammonia content and lower pH [16].

### Correlation between welfare indicators and physicochemical characteristics of litter types

Our results revealed a correlation between footpad lesions and litter types. Broilers reared on WS litter type yielded the best FPD scores compared to the other litter types, while both straw types had the worst FPD scores. These findings support those of other studies indicating a relationship between litter type and occurrence of contact dermatitis in broilers [6, 48, 49]. According to Boussaada and Ouachem [21], the type of litter affects the development of contact dermatitis (footpad, hock burn, and breast blister), with broilers reared on WS having lower dermatitis scores than those reared on other types of litter (straw, CR, and S). Sirri *et al*. [29] also reported that flocks reared on WS showed less dermatitis than those reared on straw.

We observed contact dermatitis lesions to first occur on the footpads, then the hocks, and finally the breast. Contact dermatitis may develop as a result of prolonged contact with low-quality litter. This is supported by Allain *et al*. [33], who reported that the litter quality had an impact on pododermatitis and hock burns in broiler chickens. Furthermore, poor litter quality has been considered the most important factor, leading to the appearance of lesions on the footpad or hock in broiler chickens [50] and broiler turkeys [12].

In our study, litter type was positively correlated with the litter scores (r = 0.67; p < 0.001), with the best overall scores observed in WS and S litter types, while the LQS type had the worst scores. Furthermore, severe footpad lesions (score 4) were significantly more frequent (by 60%) in broilers reared on both straw litters than other types of litter (p < 0.01). Ekstrand *et al*. [51] also found a strong correlation between the prevalence of footpad dermatitis and litter condition.

Our results showed that the footpad dermatitis was correlated with hock burn, which is consistent with prior research. Allain *et al*. [33] found a link between deep footpad lesions and black hock burns, which were then linked to breast burns. Meluzzi *et al*. [13] also found that footpad dermatitis and hock burns have a strong positive correlation (r = 0.79). On the other hand, other research found only a weak link between pododermatitis and hock burns [32, 52]. We also demonstrated that contact dermatitis observed was not correlated with any of the physicochemical characteristics (moisture, pH, or ammonia levels) of litter measured. In line with this, ammonia has previously not been found to have an effect on the severity of pododermatitis in broilers [11, 12, 17]. However, it should be highlighted that we measured litter ammonia, whereas most early research measured atmospheric ammonia [11, 12, 17, 47], thus making the effects difficult to compare. The previous studies has also found that litter pH does not affect footpad health [11, 13, 14].

On the other hand, litter moisture has previously been reported to be the main factor contributing to the appearance of footpad lesions in broilers, turkeys, and laying hens [11, 12]. Wu and Hocking [14] observed that litter wetness over 30% led to impaired footpads. Another study found a link between higher threshold moisture of 49% and a higher risk of footpad dermatitis in turkeys [53]. Discrepancies between studies could be due to differences in rearing conditions, which vary widely between countries, farms, and experimental environments.

## Conclusion

The type of litter was found to be the most important factor we examined affecting the welfare of broilers. Broilers reared on WS showed the better condition of footpads, fewer hock burns, and cleaner plumage. We found no significant difference among treatments in terms of litter pH, moisture content, or ammonia levels. Contact dermatitis observed (pododermatitis and hock burn) was not correlated to any of the physicochemical parameters. However, there were significant correlations between litter types and footpad lesions and between footpad dermatitis and hock burns.

## Authors’ Contributions

TB: Conceived and designed the study. KL and TB: Performed the study. TB and SAB: Analyzed the data. TB: Drafted and revised the manuscript. SM: Data collection. All authors have read and approved the manuscript.
